# Is access to medical education improving?

**DOI:** 10.15694/mep.2017.000185

**Published:** 2017-10-16

**Authors:** Richard Hays

**Affiliations:** 1 James Cook University

**Keywords:** undergraduate medical education, access, selection

## Abstract

This article was migrated. The article was marked as recommended.

The number of medical school places appears to be increasing faster than population growth in many parts of the world, with perhaps two main drivers. The first is the increasing population, in particular those with who are older and with chronic, complex health conditions. The second is globalisation and commercialisation of medical education, with growing numbers of fee-paying programs for applicants seeking careers in countries that offer the best career opportunities. Using Australia as an example, this paper suggests that while access to primary medical qualification programs is increasing, barriers to progress may have simply been moved to postgraduate employment and training opportunities, such that producing the workforce needed for a healthier population remains challenging.

## Background

Careers in medicine remain popular and, in most nations, admission to primary medical qualification (PMQ) programs is highly competitive. In most institutions, successful applicants must have both very high academic achievement in the recent past and demonstrated personal qualities. Academic performance has accepted and readily available measures, although their utility and meaning may be debated, and prior academic performance is a more consistent predictor of academic performance in the early years than career stages. The measurement of personal qualities is more challenging and controversial, because personal qualities may be more difficult to define and measure in young adults, particular those entering from secondary school graduation, and their predictive strength may be more relevant for later stages than for PMQ programs ^1^. Some, such as the aptitude tests used in Australia, the UK and Ireland, appear to contribute less than expected to prediction of future performance ^2- 4^, bringing in question their validity.

Gaining admission to a medical program has become a curious game of competition, chance and back-up plans for those who can demonstrate both high academic achievement and the desired personal qualities, at least as we measure these attributes. Many medical schools attract many more applicants than places, resulting in a high number of ‘losers’ in the competition, even though many of those ‘losers’ almost certainly have the ability to succeed academically and professionally. While medical schools do not necessarily like to use the term ‘rejection’, rejection is the sense that most ‘failed’ applicants have.

Avoiding rejection has become something that many applicants cannot leave to chance alone. This feeds an ‘underground’ industry that has developed around medical school admissions processes, with courses available to practice questions likely to be found in aptitude tests and to practice interview techniques for each medical school. Anecdotal reports suggest that interviewees, particularly those scheduled early in interview processes, are paid to reveal the questions and scenarios they encounter to organisations that on-sell this information to others about to be interviewed. Similarly, personal statements can be ‘coached’ and referees may be selected because they will say nice things. Admissions preparation courses are expensive, even though they are not proven to make a significant difference, except perhaps through building confidence in applicants, which may be helpful. Their cost and availability at only certain locations may however contribute to the disproportional admission of applicants from wealthier, urban and more aware families.

Medical education has recently undergone substantial expansion, with many more medical programs and scaling up of many existing programs, due to workforce shortages and mal-distributions compounded by ageing populations living with complex, chronic illnesses, and so greater need for health care. This has resulted in many more medical students and, ultimately, graduates. Using as an example Australia, which already has a high doctor:population ratio ^5^, the number of medical applicants commencing a PMQ program has approximately trebled in the last 15 years and is likely to increase further as several more medical programs are under development (see Table 1). Even accounting for the increase in population over this period, the number of commencing medical students per 100,000 people has doubled. Similar increase have occurred in the UK ^8, 9^.

Elsewhere, the expansion of medical education has been more variable, but one of the fastest growing sectors is private medical programs. Some of these are in developing nations, but appear designed for export to the developed world. These are sometimes more expensive, but are a useful back-up plan for applicants who can afford it. Again, there is an equity argument: while meritocracy is predominant in admissions processes in the developed world, capacity to pay may play a larger role elsewhere. One conclusion is that gaining admission to a PMQ program has never been easier, and may soon be even easier, particularly for applicants with the financial ability and willingness to move countries to achieve their ambition.

The impact of the expansion after graduation from PMQ programs has only recently begun to be felt. Globalisation of medical education means that recognition of PMQs, both domestically and internationally, requires substantial scaling up and processes to deal with graduates from new schools wanting to cross borders. The United States of America has acted more decisively than many jurisdictions, requiring by 2023 that all recognised PMQ programs are accredited by a national regulatory authority auspiced by the World Federation of Medical Education ^10^. Other nations are likely to follow.

Employment of all graduates is another issue, because there are already too many graduates for jobs available in some jurisdictions. Again the examples of United Kingdom and Australia are illustrative. Foundation year and Intern posts are provided because they ‘complete’ the degree and provide eligibility for full registration, but already some junior doctors are missing out on jobs because of selection failures and a lack of willingness to go where the jobs are - often less popular and attractive locations that are under-served ^8, 11^.

Further, access to postgraduate specialty training is becoming another major career-limiting step. Lack of capacity to train the increasing numbers of graduates appears to be a significant constraint. Distribution of training places to specialties increasingly reflects health workforce needs, but do not necessarily match the aspirations and interests of the graduates. Many places are currently not filled, for reasons that are not clear, although may represent a ‘gap year or two’ before embarking on the next, more focused stage. Higher cost medical education may also deter choice of needed specialties, which often attract less pay and status ^12^. While the apparent low engagement of graduates in some specialty training programs may motivate further expansion of PMQ programs, is this similar to simply pouring more liquid into a leaking bucket? Further, do we really want graduates to be ‘forced’ to work in a specialty that they do not enjoy? Does job dis-satisfaction increase the risk of error and loss of competence? These issues need exploration.

## Conclusion

Access to PMQ programs may have become easier, but that access may still favour those with the ability to pay more and to move (even internationally) to achieve their ambition. Further, while it may be easier to achieve access to a PMQ program, finding employment and training, particularly in a fulfilling and interesting career path of first choice, may become much more difficult. Finally, the more costly the medical education, the more likely may graduates be to pursue high-earning specialties that are less likely to help the world become a healthier place.

## Take Home Messages

• Access to primary medical qualification programs appears to be increasing.
• Postgraduate employment and training opportunities appear to be emerging obstacles to workforce development.
• Improved access at PMQ program level may not necessarily produce the workforce needed to make the world a healthier place.

## Notes On Contributors

Richard Hays is Professor of Remote and Rural Health at James Cook University, with current roles in postgraduate training of doctors for under-served remote and Indigenous communities.

## Declarations

Editor of AMEE MedEdPublish

## Appendices

**Table 1.** Australian population, medical student and graduate numbers 2001-2026.

	2001	2010	2016	2025
Population ^6^ (100,000)	19.4	22.3	24.1	28.8 ^a^
Domestic ^7^ students entering n, (n/100,000)	1470 (76)	2939 (132)	3215 (133)	3600 ^a^ (125)
International ^7^ students entering n, (n/100,000)	367 (19)	529 (24)	613 (25)	600 ^b^ (21)
Domestic graduates ^7^ n, (n/100,000)	1203 (62)	2259 (101)	3085 (128)	3600 ^a^ (125)
International graduates ^7^ n, (n/100,000)	113 (6)	474 (21)	484 (20)	450 ^b^ (16)

^a^ Estimate based on currently known plans

^b^ Estimate based on assuming no increase in international student enrolments.
