# Supplementary material for: Is access to medical education improving?
Source: MedEdPublish (2016). 2017 Oct 16;6:185. [Version 1] doi: 10.15694/mep.2017.000185 (PMC10885307; doi:10.15694/mep.2017.000185)
Supplement: Peer review history

# Reviewer report: Reviewer response for version 1

**DOI:** 10.21956/mep.19373.r28363

**Published:** 2017-10-21

**Authors:** Olle ten Cate

**Affiliations:** 1 UMC Utrecht

This review has been migrated. The reviewer awarded 5 stars out of 5 This is an excellent contribution highlighting novel information. The limitation is of course is de focus on one country/continent. The Netherland eg has a total enrolment number in PMQs that is controlled by the government and fully determined by an estimation of the country's own need for health care workforce. It must be realized that increase in PMQ programs without increase in postgraduate programs may evoke fierce competition among medical students.

Reviewer Expertise:

NA

No decision status is available

# Reviewer report: Reviewer response for version 1

**DOI:** 10.21956/mep.19373.r28362

**Published:** 2017-10-19

**Authors:** Ronald M Harden

**Affiliations:** 1 AMEE

This review has been migrated. The reviewer awarded 5 stars out of 5 Richard Hays highlights the dramatic increase in students admitted to study medicine illustrating this with interesting figures from Australia. He goes on to question, however, the fairness of the selection process. There is wide agreement about the attributes required of the doctor on qualification and how these can be measured. There is much less agreement, even disagreement, on the attributes expected of a student on entering medical studies and how these can be assessed. What can be done is to decide what is expected on entry for each of the exit outcome domains . We have done this for the 12 domains of the Scottish Doctor outcomes. Richard also highlights problems with pg training and matching the trainee with posts available. Perhaps as discussed at a symposium at AMEE 2017 we should admit and train from the outset, doctors in the different major specialties

Reviewer Expertise:

NA

No decision status is available

# Reviewer report: Reviewer response for version 1

**DOI:** 10.21956/mep.19373.r28364

**Published:** 2017-10-18

**Authors:** Neel Sharma

**Affiliations:** 1 Albert Einstein College of Medicine, Montefiore Medical Center

This review has been migrated. The reviewer awarded 5 stars out of 5 Thanks Richard for this valuable perspective. I do agree that there is no constant in the access process. I for one moved abroad based on funding difficulties within the UK and to explore options of interest to me and my career progression yet not fully recognised (as yet) within the umbrella of training. I find it interesting to read in the UK at least of the option to bring in physician associates to the field. I wonder what this may mean in terms of access for traditional doctors. Would it become uber competitive to receive training. Trials of such in parts of the UK have fed back that there is some competition in the admission process where PA s and doctors in training are competing for cases. Yet those calling in the shots need to enhance supply and cut costs. A fine balance.

Reviewer Expertise:

NA

No decision status is available
